# Macrofauna Associated With Submarine Groundwater Discharge Sites and a Cold Seep in Svalbard Fjords

**DOI:** 10.1002/ece3.74597

**Published:** 2026-10-23

**Authors:** Johanna Schramm, Wei‐Li Hong, Sophie ten Hietbrink, Beata Szymczycha, Jochen Knies, Arunima Sen

**Affiliations:** ^1^ Department of Arctic Biology The University Centre in Svalbard (UNIS) Longyearbyen Norway; ^2^ Department of Geological Sciences Stockholm University Stockholm Sweden; ^3^ Institute of Oceanology, Polish Academy of Sciences Sopot Poland; ^4^ Geological Survey of Norway Trondheim Norway

**Keywords:** Arctic, benthos, climate change, macrofauna, porewater freshening, Svalbard

## Abstract

Submarine groundwater discharge (SGD) is the flow of subsurface water into the ocean. To assess its impact on the Svalbard benthos, the composition of macrobenthos was investigated in relation to sediment physico‐chemical conditions. SGD has been seen to generate seep‐like ecosystems in subarctic locations through the transport of reduced chemicals such as methane. Therefore, we compared the benthos of three Svalbard fjord SGD locations to a Svalbard shelf seep location. However, contrary to our expectations, the SGD locations did not resemble the sampled cold seep. Methane was not detectable in sediment porewater, free sulfide concentrations were low, and diversity and abundance were extremely low, despite shallow water depths (40–117 m). It is possible that the combination of freshwater stress, limited chemosynthetic primary production, and high glacimarine sedimentation rates typical of glacier‐proximal fjord environments together result in an inhospitable environment for many marine benthic taxa at Svalbard fjord SGD locations. As the Arctic warms and glaciers transition from marine‐ to land‐terminating, SGD fluxes may change and the sedimentary overprint on SGD‐related ecological effects may diminish. Whether SGD will eventually emerge as an ecologically relevant process in Arctic fjord systems remains an open question, but one that will become increasingly pertinent as permafrost thaws and glaciers continue to retreat.

## Graphical abstract

This conceptual model illustrates how the balance between submarine groundwater discharge (SGD), glacimarine sedimentation, and benthic community development may change during glacier retreat in Arctic fjords. In present‐day marine‐terminating systems, high sedimentation and physical disturbance may mask the ecological effects of SGD. As glaciers retreat, reduced sedimentation may allow SGD‐related ecological effects to become more apparent, potentially supporting increasingly diverse benthic communities.

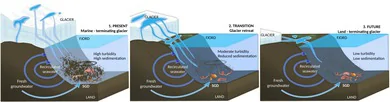

## 1 Introduction

Submarine groundwater discharge (SGD) is the discharge of subsurface fluids across the land‐ocean interface (Moore et al. 2008). SGD includes either freshwater or recirculated seawater, or a mixture of both, driven by hydraulic head gradients and circulation through a subterranean estuary (Diak et al. 2023; Moore 1999; Taniguchi et al. 2002; Moore et al. 2008; Lecher and Mackey 2018). SGD locations can occur in a variety of coastal environments around the world. They can, for example, be found at estuaries, lagoons, islands, mangroves, salt marshes or Arctic fjords (Santos et al. 2021; Sekar et al. 2022; Lecher 2017). However, SGD is not limited to coastal areas and can occur at considerable distances from the coastline, when confined aquifers beneath the continental shelf outcrop on the seafloor (Kim et al. 2022; Hoffmann et al. 2023; George et al. 2020; Ten Hietbrink et al. 2025). SGD can flush chemicals such as methane and nutrients like phosphorus, inorganic nitrogen and silicate out of the sediment and transport them into the ocean (Bussmann et al. 1999; Dando 2010; Johannes 1980; Lecher 2017; Rodellas et al. 2015). Therefore, the geochemical cycles of some elements may be strongly influenced by SGD (Sekar et al. 2022). Furthermore, the discharge of groundwater into the ocean can cause a modification of pH, salinity and temperature (Szymczycha et al. 2023), the accumulation of organic matter (Szymczycha et al. 2025), and the delivery of elevated concentrations of metals (Abdelwahab et al. 2025). All these factors can impact marine ecosystems (Rahman et al. 2022; Lecher 2017; Sekar et al. 2022).

Organisms that inhabit SGD locations must be able to withstand, grow, and maintain productivity while being exposed to rapid fluctuations in nutrient availability and changing environmental conditions (Santos et al. 2021). Freshened sediment porewater favors euryhaline organisms adapted to low salinity or tolerant of a wide range of salinity levels (Dale and Miller 2008). Moreover, nutrients transported by SGD and released from the sediment can enhance primary production in both the euphotic zone and the benthos, depending on water depth and light availability (Cho et al. 2018; Taniguchi et al. 2019). However, the extent of this enhancement depends on factors such as water column mixing and nutrient fluxes (Taniguchi et al. 2019; Liu et al. 2017). When it does occur, increased phytoplankton growth and photosynthetic primary production lead to a higher supply of organic matter to the seafloor, which can enhance benthic abundance and biomass (Lecher and Mackey 2018; Crespo and Pardal 2020). On the other hand, nutrient enrichment from SGD can cause eutrophication, leading to low‐oxygen conditions in the water column, resulting in a shift towards benthic species that can tolerate hypoxic environments (Diaz 2016; Dai et al. 2023).

Besides nutrient enrichment, SGD locations can additionally be associated with methane that is transported with groundwater (Hong et al. 2019; Szymczycha et al. 2025; Ten Hietbrink et al. 2025). In Arctic regions such as Svalbard, this methane can originate from microbial degradation of organic matter in permafrost soils or from deeper thermogenic sources (Birchall et al. 2023). Meltwater from glaciers and seasonally thawed permafrost mobilizes these reduced compounds (Kleber et al. 2025), which may then be carried along subsurface flow pathways to the sea. Upon discharge at the seafloor, methane can fuel chemosynthetic microbial communities, either directly, or through the generation of sulfide via its anaerobic oxidation coupled to sulfate reduction (Knittel and Boetius 2009). Chemosynthetic microbes can either be free‐living and form microbial mats on the sediment surface, or they can occur within or on organisms in symbiotic relationships (Åström et al. 2020; Gebruk et al. 2003; Pimenov et al. 2000; Lösekann et al. 2008). This results in autochthonous local primary production at the seabed at seep locations, with chemosynthetic microbes at the base of the food chain. Therefore, cold seeps often display high biomass and abundance (Åström et al. 2020; Wang et al. 2023), but low diversity due to the toxicity of sulfide (Bagarinao 1992; Vismann 1991; Cordes et al. 2010). The transport of methane can thus result in SGD locations resembling cold seeps, which has indeed been observed in subarctic regions (Sen et al. 2019; Hong et al. 2019).

In this study, the term SGD refers specifically to the discharge of fresh or freshened groundwater, as indicated by the freshening of the sediment porewater. Whether SGD generates high‐abundance seep‐like ecosystems in high Arctic fjords remains an open question and forms the basis for this study. We hypothesized that Arctic SGD locations would resemble cold seep locations at similar water depths, with elevated abundance and biomass due to chemosynthetic primary production driven by SGD‐derived methane transport and associated sulfide generation. To test this, we examined and quantified the macrobenthos at SGD locations in the fjords of the high Arctic archipelago of Svalbard and compared these with a cold seep on the adjacent shelf at similarly shallow water depths.

## 2 Methods

All sampling took place in March 2023 onboard the research vessel *Kronprins Haakon* as part of the Arctic SGD project. In 2022, freshened sediment porewater was detected at a number of locations in Svalbard fjords (Kim et al. 2022; Saghravani et al. 2024; Szymczycha et al. 2025). For this study, we concentrated on three locations where the strongest signs of freshening were observed, which were in the fjords of Tempelfjorden and Hornsund (Figure 1). Both fjords have land‐ and marine‐terminating glaciers, which may contribute to SGD through subglacial recharge, whereby meltwater infiltrates beneath the glacier and recharges the underlying groundwater system (Lecher 2017). In Tempelfjorden, we sampled two locations. One was located in the inner part of the fjord (T‐SGD; 78.4 m water depth) and the second was the so‐called Murdoch Hole (M‐SGD; 39.5 m water depth), where strong localized currents prevent stable sea ice formation during winter (Johansen 2000; Morozov et al. 2019). In Hornsund, the southernmost fjord of Svalbard, SGD was observed near several active tidewater glaciers in the inner basin (Kim et al. 2022; Saghravani et al. 2024). The sampling location (H‐SGD) was located at a depth of 117 m (Table 1, Figure 1).

**FIGURE 1:**
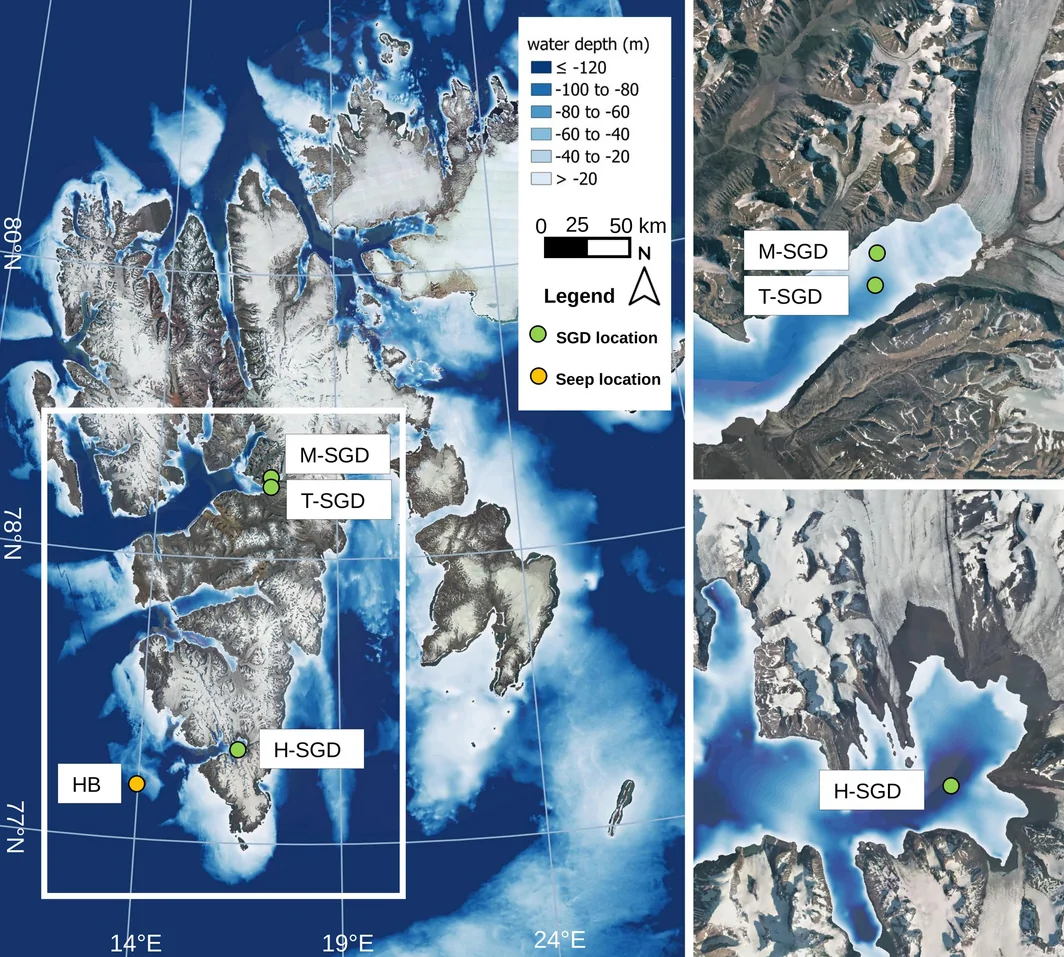
Map of the sampling locations. The main panel on the left shows Svalbard and an overview of all the sampling locations in the different fjords. The two panels on the right show close‐ups of Tempelfjorden (top) and Hornsund (bottom) with the sampling sites. Green circles indicate SGD sites (H‐SGD, Hornsund SGD site; M‐SGD, Murdoch Hole; T‐SGD, Tempelfjorden SGD site) and the yellow circle indicates the cold seep site (HB, Hornsundbanken). Bathymetric data from the International Bathymetric Chart of the Arctic Ocean (Jakobsson et al. 2024). Basemap: Norwegian Polar Institute (npolar.no).

**TABLE 1:** Overview of the sampling sites. The first column refers to the full name of each sampling location and the second column refers to the short name used in the text. Lat (°N) and Long (°E) indicate the latitude and longitude of each site in decimal degrees respectively. Device: Macrobenthos refers to the corer used for macrobenthos sampling, and Device: Porewater refers to the corer(s) used for sediment porewater sampling. Depth (m) represents the water depth in meters. Salinity refers to bottom water salinity in psu and temperature refers to bottom water temperature in °C.

Location and name of site	Short name	Lat (°N)	Long (°E)	Device: macrobenthos	Device: porewater	Depth (m)	Salinity (psu)	Temperature (°C)
Tempelfjorden‐SGD	T‐SGD	78.41	17.14	GEMAX	Push core	78.4	34.6	−0.96
Murdoch Hole‐SGD	M‐SGD	78.43	17.14	GEMAX	Push and gravity core	39.5	32.2	−1.26
Hornsund‐SGD	H‐SGD	77.00	16.47	Push core	Push and gravity core	117	34.6	0.17
Hornsundbanken seep microbial mats	HB‐mats	76.79	14.16	Push core	Push core	126	34.7	0.71
Hornsundbanken regular sediment	HB‐reg	76.79	14.16	Push core	Push core	126	34.7	0.71

On the shelf adjacent to the Hornsund fjord, multiple gas flares have been observed acoustically, indicating the presence of a cold seep (Figure 1) (Mau et al. 2017). In March 2023, we also detected hydroacoustic flares at this location and visually confirmed gas seepage using the remotely operated vehicle (ROV) Ægir 6000. This seep on the Hornsund shelf, Hornsundbanken (HB, 126 m water depth), therefore served as a shallow‐water Svalbard cold seep for comparison with the SGD locations in the fjords.

### 2.1 Sampling and Macrobenthos Processing

Bottom water temperature and salinity were recorded using a Sea‐Bird SBE 911plus CTD system equipped with temperature and conductivity sensors. At the two Tempelfjorden locations, T‐SGD and Murdoch Hole (M‐SGD), two replicate sediment cores (9 cm in diameter) were collected directly from the research vessel using a GEMAX corer (Table 1). At the Hornsund SGD location (H‐SGD), three replicate push cores (10 cm in diameter) were taken with the remotely operated vehicle (ROV) Ægir 6000 (Table 1). Due to poor visibility and high turbidity, push cores at H‐SGD were taken directly in front of the ROV. Better visibility at the Hornsundbanken seep allowed for more precise sampling; therefore, at this location, two replicate push cores (10 cm in diameter) were taken with the ROV from each of two microhabitats: microbial mats (HB‐mats) and regular seep sediment not covered with microbial mats (HB‐reg) (Table 1, Figure 5).

Sediment cores were sieved over a 0.5 mm mesh and immediately preserved in 4% buffered formaldehyde. In the lab, samples were transferred to 70% ethanol. The macrobenthos was sorted under a *Leica MZ16 A* stereomicroscope and identified to the lowest taxonomic level, which was usually species level. Species names were verified with the World Register of Marine Species (WoRMS Editorial Board 2025). The number of individuals for each taxon or species was then counted, and wet weight determined after blotting on a scale that resolves to 0.001 g. Damaged specimens and polychaetes lacking a head, which could not be reliably identified, were not assigned to a taxon and therefore not included in abundance counts but were included in biomass measurements. Due to the two different collection methods used (push cores vs. GEMAX cores), both abundance and biomass were standardized to m^2^.

### 2.2 Porewater Analyses

Gravity cores and push cores were taken for collecting sediment porewater to characterize sediment geochemistry. Push cores were taken at all four locations, and at Murdoch Hole (M‐SGD) and Hornsund (H‐SGD), additionally one gravity core was taken (see Table 1 for overview of sampling efforts across the different locations). Porewater samples were collected by inserting rhizons (Seeberg‐Elverfeldt et al. 2005) at predefined intervals into the sediment cores. Gravity cores were sampled at 20 cm intervals, whereas push cores were sampled at 2 cm intervals. However, porewater was not always retrieved from every interval.

To determine total hydrogen sulfide concentrations (∑H_2_S), a 0.5 mL aliquot of porewater was mixed with an equal volume of saturated Zn(OAc)_2_ solution and subsequently frozen onboard to stabilize dissolved hydrogen sulfide (Hong et al. 2017). Methane concentrations were determined from sediment samples using a headspace equilibration technique, with gas analysis performed by gas chromatography (SRI 8610C) following standard procedures.

In the laboratory, dissolved sulfide (∑H_2_S = H_2_S + HS^−^ + S^2−^) was measured through titration (USGS Method I‐3840 (Fishman and Friedman 1989)) with a detection limit of 20 μM. Briefly, 0.2 mL of I_2_ solution, 0.05 mL of starch solution, and 0.05 mL of 4 M HCl were sequentially added to the sample. The zinc sulfide in the porewater sample reacts with I_2_ to form I^−^ in acid solution. The remaining I_2_ is then titrated by sodium thiosulfate (Na_2_S_2_O_3_·5H_2_O). The I_2_ solution was made by dissolving 3.53 g of resublimed I_2_ in 1 L of KI solution that was prepared by dissolving 6 g of iodate‐free KI into double‐distilled deionized water (18.2 MΩ·cm, Millipore). The I_2_ solution was then standardized with sodium thiosulfate of three concentrations (0.0109 N, 0.00109 N, and 0.000109 N) to achieve better precision. Starch solution was made by first dissolving 1 g starch powder in ca. 5 mL of double‐distilled deionized water by vigorous shaking in a 15 mL Falcon tube. The cold starch solution was then poured into boiling water and allowed to boil for ca. 3 min. Upon cooling, it was poured into a 50 mL Falcon tube. The starch solution was renewed weekly. The sodium thiosulfate titrant of different concentrations was added sequentially to the samples or unreacted I_2_ solution until the purple color faded away. The concentration of I_2_ was checked every three to four samples and renewed when the solution was oxidized too much. Concentrations of total hydrogen sulfide were then calculated from the concentrations of residual I_2_ that had not reacted with sulfide. Dissolved Fe^2+^ concentrations were determined by the Inductively Coupled Plasma Optical Emission Spectroscopy (ICP‐OES) at the Department of Geological Sciences, Stockholm University. Sulfate and chloride concentrations were measured using a Dionex ICS‐1100 ion chromatograph equipped with an AS‐DV autosampler and an IonPac AS23 column (Hong et al. 2019).

### 2.3 Statistical Analyses

Unless stated otherwise, statistical analyses were performed using PAST v.4.03 (Hammer et al. 2001). Diversity indices were calculated based on abundance and biomass standardized to m^2^, including taxa richness, Shannon index, Pielou's evenness, and the Chao 1 index (Table 2). Abundance data were Hellinger‐transformed (Legendre and Gallagher 2001) prior to community‐level analyses. A UPGMA cluster analysis based on Bray–Curtis dissimilarities (Bray and Curtis 1957) of Hellinger‐transformed abundance data was conducted to visualize grouping patterns among the samples (Figure 4). To test whether observed clustering was significant, a one‐way ANOSIM (Analysis of Similarities) test was performed. In addition to the cluster analysis, a non‐metric multidimensional scaling (nMDS) ordination was conducted based on the same Bray–Curtis dissimilarities to further visualize similarities among the samples (Figure 4). Additionally, a SIMPER (Similarity Percentage) analysis was performed to identify which taxa contributed the most to the observed differences between the seep and SGD samples (Table 3). The nMDS ordination was computed in R v.4.3.2 (R Core Team 2023) using the vegan package (Oksanen et al. 2022). Porewater concentration profiles (Figure 2) were visualized in R with the ggplot2 package (Wickham 2016).

**TABLE 2:** Overview of benthic community metrics for all sampling stations. Abundance and biomass values are standardized to 1 m^2^.

Site	Taxa richness	Abundance (ind./m^2^)	Biomass (g/m^2^)	Shannon index	Pielou's evenness	Chao 1
T‐SGD1	6	1415	494.268	1.67	0.94	14
T‐SGD2	5	1729	21.252	1.42	0.88	6
M‐SGD1	5	1100	9.244	1.48	0.91	11
M‐SGD2	8	2043	14.272	1.99	0.96	10.7
H‐SGD1	6	2674	5.552	1.30	0.73	12
H‐SGD2	2	1146	8.812	0.35	0.50	2
H‐SGD3	3	1655	6.724	0.69	0.63	3.5
HB‐mats1	12	5857	1801.900	1.90	0.76	16.2
HB‐mats2	8	3820	32.004	1.59	0.77	16
HB‐reg1	9	4711	429.632	1.97	0.90	9.3
HB‐reg2	10	4584	2295.036	1.95	0.85	12.3

**TABLE 3:** Results of the SIMPER analysis comparing SGD and seep communities. Contrib. (%) indicates the percentage contribution of each taxon to the average Bray–Curtis dissimilarity between the two groups, and Cumulative (%) represents the cumulative contribution. Mean (SGD) and Mean (Seep) denote the mean Hellinger‐transformed abundance of each taxon in the respective sample group. Taxa are listed until a cumulative contribution of at least 70% is reached.

Taxon	Contrib. (%)	Cumulative (%)	Mean (SGD)	Mean (Seep)
*Aphelochaeta marioni*	12.05	12.05	0.565	0.345
*Yoldiella propinqua*	7.44	19.50	0.110	0.272
*Leucon nasica*	6.15	25.65	0.202	0
*Notoproctus oculatus*	5.78	31.43	0	0.181
*Cossura pygodactylata*	5.72	37.14	0.0708	0.137
*Lumbrineris mixochaeta*	4.75	41.89	0.452	0.585
*Robertsonites tuberculatus*	4.50	46.39	0.150	0
*Naineris quadricuspida*	4.37	50.76	0.0792	0.130
*Yoldiella lenticula*	4.15	54.92	0	0.130
*Chaetoderma nitidulum*	3.90	58.82	0	0.123
*Yoldiella intermedia*	3.88	62.70	0	0.116
*Annelida* sp.	3.44	66.14	0	0.104
*Ennucula tenuis*	2.88	69.03	0.0476	0.0638
*Ctenodiscus crispatus*	2.64	71.67	0	0.0828

**FIGURE 2:**
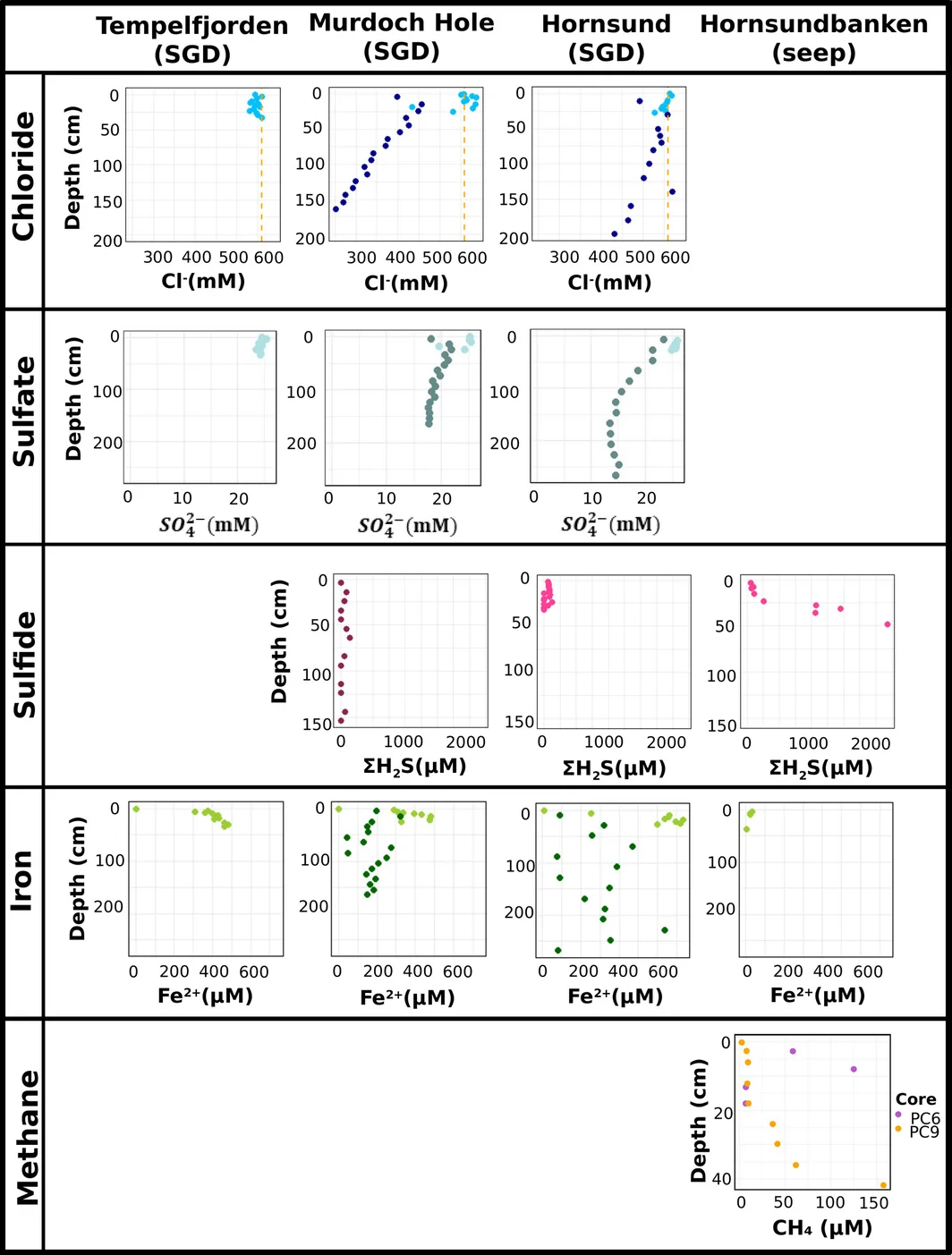
Downcore profiles of sediment porewater chloride, sulfate, sulfide, iron, and methane concentrations at the study sites. The orange dashed line represents seawater chloride concentration at a salinity of 35 (Hong et al. 2017; Millero et al. 2008). Darker colors represent samples from gravity cores and lighter colors represent samples from push cores. For methane, the two colors represent the two different push cores. Sulfide values below the detection limit (20 μM) are plotted as 0. Sulfide concentrations at Tempelfjorden and methane concentrations at all SGD locations were below the detection limit and are therefore not shown.

## 3 Results

### 3.1 Environmental Conditions

Bottom water temperature varied slightly across locations, with the lowest values recorded at Murdoch Hole (M‐SGD) (−1.26°C), whereas the highest temperature was measured at the Hornsundbanken seep (0.71°C). Bottom water salinity was the lowest at Murdoch Hole (32.2 psu) and the highest at the Hornsundbanken seep (34.7 psu) (Table 1).

Chloride concentrations from push cores at all SGD locations (approximately 40 cm recovery) were close to seawater concentrations (approximately 560 mM at a salinity of 35; Hong et al. 2017; Millero et al. 2008; Figure 2). Measurements from gravity cores (at Murdoch Hole and Hornsund) showed further decreases in chloride concentration with increasing depth, which was particularly pronounced at Murdoch Hole (down to 251 mM, Figure 2). Sediment porewater methane concentrations at all SGD locations were below the detection limit of the headspace gas chromatography method (~0.09 μM). At the Hornsundbanken cold seep, methane concentrations generally increased with sediment depth, most clearly in core PC9, where concentrations rose from near‐detection levels close to the sediment–water interface to approximately 158 μM at 42 cmbsf (Figure 2). Sulfide concentrations were below the detection limit (20 μM) at Tempelfjorden and remained low at Murdoch Hole and the Hornsund SGD site, where they did not exceed 144 μM. In contrast, at the Hornsundbanken cold seep, sulfide increased with depth, reaching millimolar concentrations (Figure 2). Sulfate concentrations exhibited a general decrease with depth at all the SGD locations. At Murdoch Hole, the gradual decline paralleled the concurrent chloride decrease. At Hornsund, however, sulfate declined more sharply within the upper part of the core and then remained comparatively stable with depth, whereas chloride continued to decrease more gradually over the same interval (Figure 2). Iron concentrations were consistently high at all SGD locations, with the highest concentrations at the Hornsund SGD location (H‐SGD). On the other hand, iron was almost entirely absent downcore at Hornsundbanken cold seep (Figure 2).

### 3.2 Community Composition

A total of 232 individuals representing 33 taxa across five phyla were recorded. Values are given as mean ± SD Mean abundance was 1680.4 ± 549.9 ind./m^2^ (*n* = 7) among the SGD samples, which was roughly 3 times lower than what was observed among the seep samples (mean abundance = 4742.8 ± 840.6 ind./m^2^, *n* = 4). Biomass was on average 14 times higher among the seep samples (mean biomass = 80.0 ± 182.7 g/m^2^, *n* = 7 among the SGD samples vs. 1139.6 ± 1080.8 g/m^2^, *n* = 4 among the seep samples). Taxa richness was on average twice as high among the seep samples (9.75 ± 1.71 taxa, *n* = 4) than among the SGD samples (5.0 ± 2.0 taxa, *n* = 7). Similarly, Shannon diversity was greater among the seep samples (1.85 ± 0.18, *n* = 4) than among the SGD samples (1.27 ± 0.57, *n* = 7), while evenness was similar across samples from both environments (seep samples: 0.82 ± 0.06, *n* = 4; SGD samples: 0.79 ± 0.18, *n* = 7) (Table 2). The Chao 1 value showed higher estimated richness among the seep samples, with a mean value of 13.4 (mean taxa richness 9.8) compared to 8.5 (mean taxa richness 5.0) among the SGD samples.

Annelida was the most abundant phylum across all samples, accounting for 75% of all individuals and was particularly dominant among the Hornsund SGD samples, where it comprised 95% of the total number of individuals (Figure 3). Mollusca was the second most abundant phylum but was absent from the Hornsund SGD samples. Arthropoda ranked third in abundance and was notably absent from samples from the Hornsundbanken seep (Figure 3).

**FIGURE 3:**
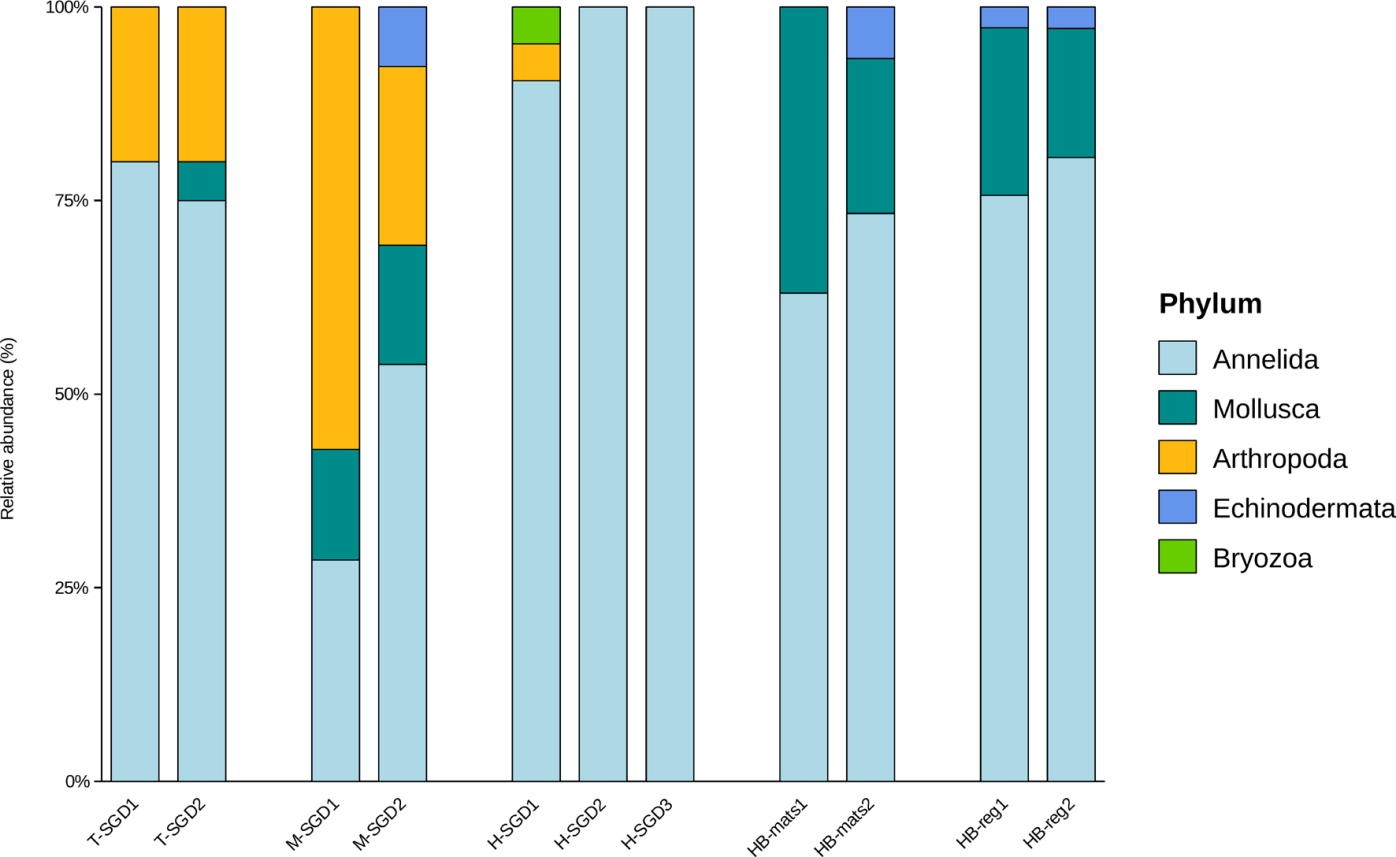
Relative abundance of macrobenthos phyla in each sample, grouped by location (HB‐mats, Hornsundbanken microbial mats; HB‐reg, Hornsundbanken seep sediment without microbial mats; H‐SGD, Hornsund SGD; M‐SGD, Murdoch Hole; T‐SGD, Tempelfjorden SGD).

The Hornsundbanken (HB) seep samples separated from the SGD samples (Figure 4) and based on the ANOSIM, this separation was significant (global *R* = 0.5185, *p* = 0.0091). According to the SIMPER analysis, the polychaete *Aphelochaeta marioni* and the bivalve *Yoldiella propinqua* contributed most to the dissimilarity between the SGD and seep communities (12.05% and 7.44% contribution, respectively), though both occurred in both types of samples. Among taxa with a smaller but still notable contribution, the cumacean *Leucon nasica* (6.15%) was found exclusively among the SGD samples and the polychaete *Notoproctus oculatus* (5.78%) exclusively among the seep samples, while the polychaete *Cossura pygodactylata* (5.72%) was present among both types of samples (Table 3).

**FIGURE 4:**
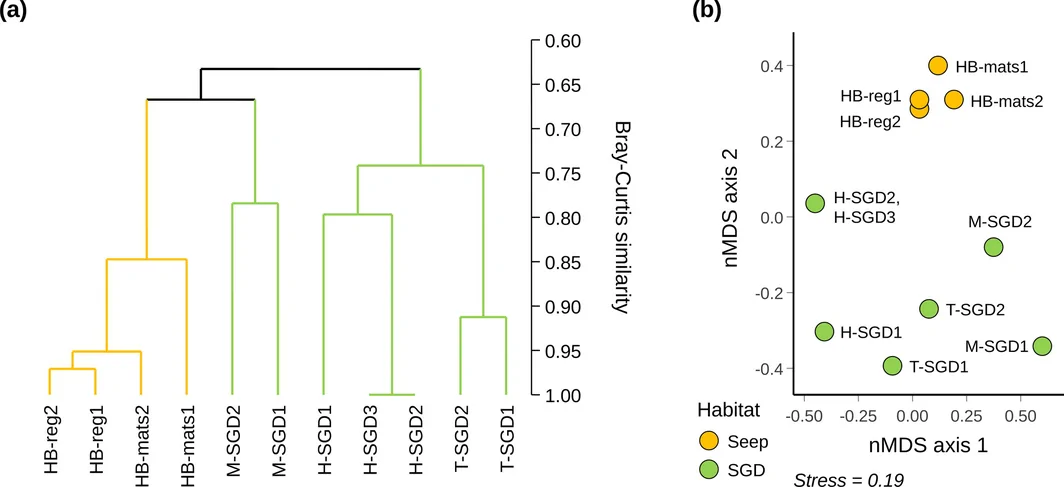
(a) UPGMA cluster analysis and (b) non‐metric multidimensional scaling (nMDS) ordination based on Bray–Curtis dissimilarities of Hellinger‐transformed abundance data (stress = 0.19). Green indicates SGD samples and yellow indicates Hornsundbanken seep samples.

## 4 Discussion

Submarine groundwater discharge plays a key role in coastal ecosystems, influencing nutrient fluxes and benthic community structure (Santos et al. 2021; Grzelak et al. 2018; Kotwicki et al. 2014; Yu et al. 2024). Although SGD can generate cold seep environments in subarctic areas due to methane transport supporting chemosynthesis‐based communities (Sen et al. 2019), we did not observe seep‐like communities at the Svalbard SGD locations we sampled. We expected the abundance and biomass in the samples from our SGD study locations to be as high as those in the samples from our cold seep location, assuming that chemosynthesis was occurring at the SGD locations due to the presence of methane and sulfide. Instead, samples from the SGD study locations exhibited low biomass, diversity, and taxa richness. A key assumption of our hypothesis was that methane is transported with SGD and present in sediment porewater at the SGD study locations. However, sediment methane concentrations were below detection limits at the SGD locations. This suggests that chemosynthesis via methane oxidation is likely not taking place or is very limited. Furthermore, the absence of detectable methane suggests that the anaerobic oxidation of methane (AOM) is likely very limited as well, preventing the generation of appreciable amounts of sulfide, which, with methane, is a key energy source for chemosynthetic organisms (Jenkins 2011; Gao et al. 2022). Indeed, sulfide concentrations at our SGD locations were low or below the detection limit and more than an order of magnitude lower than at the Hornsundbanken seep, which suggests that chemosynthesis via sulfide oxidation does not occur there to a large extent either (Figure 2). Therefore, it is likely that chemosynthesis‐based primary production is limited or negligible at the studied SGD locations, making them, at the ecosystem level, very different from cold seep systems.

It should be noted that our low sulfide measurements alone do not necessarily mean that sulfide is not generated through AOM. We measured high concentrations of iron at the SGD locations (Figure 2). Sulfide reacts with iron to form insoluble iron sulfides, which would reduce the concentration of free sulfide (Rickard and Luther 2007) being available for chemosynthesis (Hong et al. 2020). Therefore, even if AOM is taking place and sulfide is being generated at the SGD locations, the iron content of porewater there could nevertheless result in very low concentrations of free sulfide available for chemosynthetic processes (Pal et al. 2018; McKenzie et al. 2024). The low sulfide levels at the SGD study locations could subsequently be the result either of low rates of AOM or AOM‐generated sulfide precipitating as iron sulfide. Regardless, there would be an absence or limitation of free sulfide and subsequently, of thiotrophy, or chemosynthesis via sulfide oxidation. Microbial mats would not be very prevalent and furthermore, faunal groups with sulfide‐oxidizing symbionts would not be present. At Arctic seeps, all symbionts in chemosymbiotrophic faunal species that have been identified so far have been thiotrophic and not methanotrophic (Sen et al. 2023, 2018; Lösekann et al. 2008; Lee, Noh, et al. 2019; Lee, Kim, et al. 2019). Therefore, a lack of free sulfide at the SGD locations would prevent the settlement and recruitment of typical Arctic chemosymbiotrophic fauna. This is consistent with the species identified at the SGD locations: groups such as siboglinid worms and thyasirid bivalves which are present or even dominate high Arctic seep systems were absent at our SGD locations.

In contrast, we measured high concentrations of sulfide in sediment porewater from push cores taken at the Hornsundbanken seep (Figure 2). We also measured much lower iron concentrations at Hornsundbanken compared to the SGD locations. Conditions for sulfide‐based chemosynthesis are therefore likely more favorable at Hornsundbanken than at the SGD locations. This is supported by the presence of microbial mats on the sediment surface and indeed, microbial mats were one of the two microhabitats sampled at Hornsundbanken (Figure 5). It is possible that the low diversity, abundance, and biomass among the SGD sites could be due to chemosynthesis and seafloor‐based primary production being highly limited, resulting in lower food availability at those locations in comparison with the Hornsundbanken seep.

**FIGURE 5:**
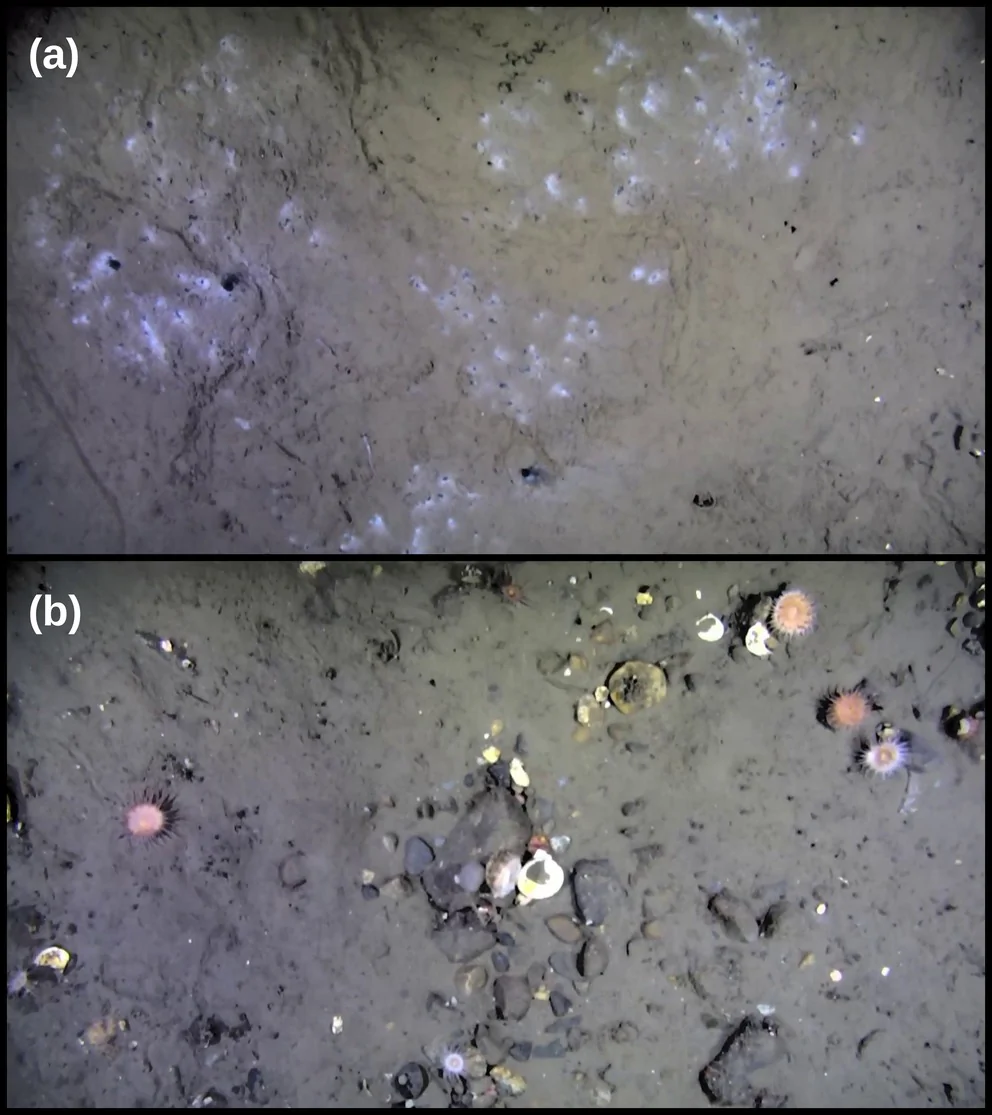
Substrate types observed at the Hornsundbanken cold seep: Microbial mat (a) and regular sediment (b).

Porewater chloride concentrations at the SGD locations were lower than seawater, most clearly in the gravity cores from Murdoch Hole and Hornsund (Figure 2), indicating freshwater input from SGD. At Tempelfjorden (T‐SGD), no gravity core was available, and chloride concentrations in the shallower push‐core profile remained close to seawater values. SGD‐related porewater freshening of meteoric origin has previously been documented in Tempelfjorden (Szymczycha et al. 2025), which is why this location was sampled, but it was not detected in our short cores at the time of sampling. The porewater evidence for SGD at T‐SGD is therefore weaker than at the other two SGD locations, which should be considered when interpreting results from this location. Porewater freshening at the SGD locations may represent an additional stressor contributing to the low diversity and abundance observed among the SGD samples, as many marine organisms are adapted to stable salinity conditions and may be sensitive to fluctuations (Weslawski et al. 2011). Freshwater alters osmotic balances and may restrict the presence of stenohaline species (Szymczycha and Pempkowiak 2016). Species such as the polychaetes *Lumbrineris mixochaeta* and *Aphelochaeta marioni* were the most abundant across all SGD and seep samples alike. Weslawski et al. (2012) identified *Lumbrineris mixochaeta* as one of the most widely distributed species in European Arctic fjord benthos, and *Aphelochaeta marioni* is also an abundant species around Svalbard (Weslawski et al. 2012; Kedra et al. 2011). It is possible that these species have a wide tolerance range regarding salinity, which could contribute towards their wide distribution around Svalbard, including at our SGD locations. Varying salinity has been described as an important driver of changes in the structure of macrobenthos due to physiological barriers that limit spatial distribution (Chikina et al. 2023). For example, community composition has been seen to vary along the estuarine and freshwater gradient of Ob Bay in northern Russia (Chikina et al. 2023). Similar patterns of shifts in community composition in response to freshwater influence have furthermore been seen in other locations as well (Rutger and Wing 2006; Laurino and Turra 2021). The differences in community composition and number of taxa, diversity, etc. between our seep samples and SGD samples could similarly be the result of exposure to freshwater and variable salinity at the SGD locations.

Beyond salinity fluctuations, high glacimarine sedimentation rates in the inner fjord basins may represent an additional environmental control on benthic settlement and diversity at our SGD locations. Svalbard fjords with active tidewater glaciers are known to receive substantial clastic sediment input via meltwater plumes, particularly during the spring and summer melt season (Görlich et al. 1987; Streuff et al. 2015). At Hornsund, sediment accumulation rates of up to 35 cm per year have been documented in ice‐proximal zones, with accumulation rates following an exponential decay away from the glacier front (Görlich et al. 1987; Majewski et al. 2009). Furthermore, high porewater iron concentrations at our SGD locations suggest substantial glaciogenic particle input, which is characteristic of glacier‐proximal settings in Svalbard fjords (Wehrmann et al. 2014).

High sedimentation rates of inorganic material near glacier fronts dilute organic particles on the seafloor and promote the burial of fauna. Suspension feeders and filter feeders are particularly negatively affected through clogging of feeding organs, while more motile taxa tend to persist under these conditions (Wlodarska‐Kowalczuk et al. 2005; Jordà‐Molina et al. 2023). A general decline in benthic diversity from outer to inner fjord basins in Svalbard is well documented and widely attributed to gradients of glacial sedimentation (Görlich et al. 1987; Wlodarska‐Kowalczuk et al. 2005). At Billefjorden, another Svalbard fjord sampled at similarly shallow depths (20–60 m), mean taxa richness ranged from 14.5 to 35.8 taxa per 0.1 m^2^ grab, with the lowest value at the marine‐terminating glacier location, the most sediment‐ and turbidity‐impacted site (van der Kamp et al. 2025). In comparison, we found 2–8 taxa per core (0.006–0.008 m^2^) at our SGD locations. Although richness is not directly comparable because of the different sampled areas and sieve mesh sizes (1.0 vs. 0.5 mm), this suggests that glacimarine sedimentation alone may not fully explain the low richness at our SGD locations.

Taken together, freshwater discharge and high glacimarine sedimentation rates might each limit which taxa can persist at our SGD locations, for example through burial stress that restricts benthic colonization (Görlich et al. 1987; Wlodarska‐Kowalczuk et al. 2005), and it is likely that both stressors act simultaneously at these locations. This combination, together with the limited chemosynthetic primary production resulting from the near‐absence of methane and sulfide at the SGD sites, could explain why abundance and biomass were so much lower in our SGD samples than in the seep samples. Our SGD samples differed markedly from the seep samples in biomass, diversity, and porewater geochemistry, and together these differences suggest that SGD locations are not analogous to the cold seep location. The cold seep habitat was represented by a single location (Hornsundbanken), sampled across two microhabitats, and was therefore not independently replicated, whereas the three SGD locations are geographically distinct locations rather than replicates of a single SGD habitat type. Nonetheless the differences between them are striking and the abundance and biomass of the SGD samples are low even when compared to other non‐seep fjord systems (van der Kamp et al. 2025). Although the GEMAX cores (9 cm in diameter, ≈64 cm^2^) sampled a slightly smaller area than the push cores (10 cm in diameter, ≈79 cm^2^), abundance and biomass were standardized to m^2^, and this difference of about 20% in sampled area is unlikely to explain the much lower abundance, biomass and richness among the SGD samples. These differences are therefore unlikely to result from the sampling methods but rather, as we posit, are driven by local environmental conditions. In short, we suggest that the absence of detectable methane and the low concentrations of free sulfide limit chemosynthetic primary production and likely preclude chemosymbiotrophic fauna, which are otherwise a key driver of elevated biomass at Arctic seeps (Wang et al. 2023). Limited seafloor‐based primary production, combined with porewater freshwater intrusion (and subsequently variable salinity) as well as potentially high glacimarine sedimentation, likely makes the SGD locations sampled here highly stressful environments, resulting in low biomass and diversity.

As climate change accelerates glacial melt and permafrost thaw, SGD fluxes in polar regions may change, with increases expected where permafrost thaw enhances subsurface connectivity (Diak et al. 2023). At the same time, intensified glacial melt will drive higher sediment delivery to inner fjord basins, which could potentially overprint SGD‐related ecological effects (Figure 6). As glaciers across the Arctic transition from marine‐ to land‐terminating, SGD may progressively emerge as an ecologically relevant process. Whether SGD fluxes will eventually become ecologically detectable against this sedimentary background remains an open question as Arctic fjord systems continue to change.

**FIGURE 6:**
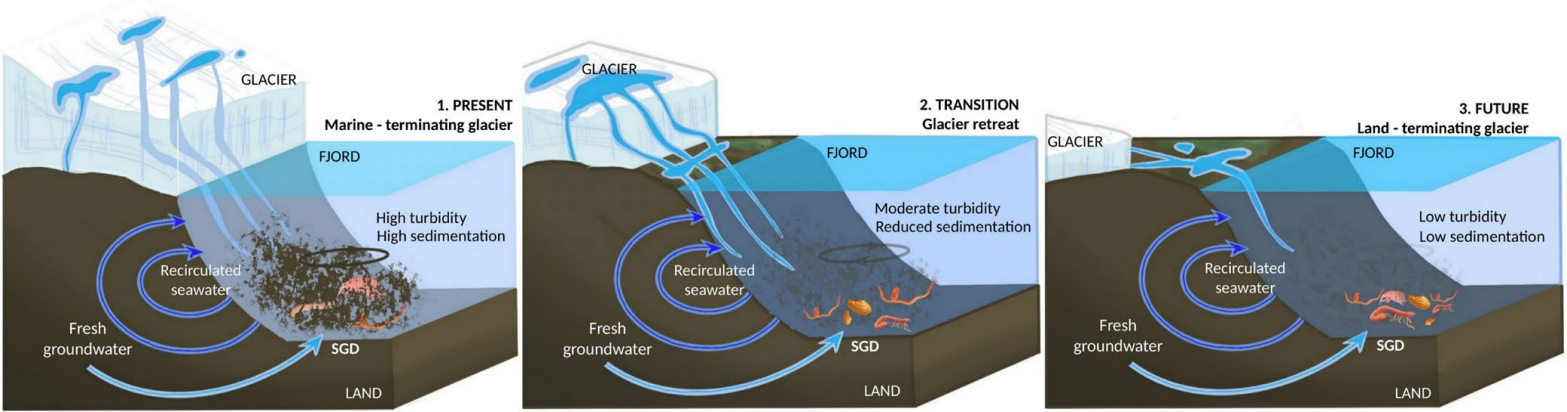
Conceptual hypothesis illustrating how glacier retreat may influence SGD‐related ecological effects in Arctic fjords, shown across three stages: (1) present‐day marine‐terminating glacier, (2) glacier retreat (transition), and (3) future land‐terminating glacier. SGD is depicted as the combined discharge of fresh and recirculated groundwater at the fjord seafloor, driven by hydraulic gradients and groundwater circulation independent of glacier presence. This discharge, and the width of the SGD arrow, therefore remain the same across all three panels. What changes between panels is the turbidity and sedimentation in the water column above the seafloor, driven by glacial meltwater entering the fjord separately from SGD, which decreases as the glacier retreats. Under high turbidity and sedimentation (present‐day), benthic fauna are illustrated as sparse and partly obscured. As sedimentation decreases (transition, future), a progressively more diverse and abundant benthic community is depicted. Even further in the future, enhanced infiltration of meltwater, precipitation, and surface runoff into subsurface flow systems may further strengthen fresh groundwater input and SGD.

## Author Contributions

**Johanna Schramm:** data curation (lead), formal analysis (lead), visualization (lead), writing – original draft (lead). **Wei‐Li Hong:** formal analysis (lead), funding acquisition (lead), investigation (lead), writing – review and editing (supporting). **Sophie ten Hietbrink:** formal analysis (lead), investigation (lead), writing – review and editing (supporting). **Beata Szymczycha:** formal analysis (supporting), funding acquisition (lead), investigation (supporting), writing – review and editing (supporting). **Jochen Knies:** formal analysis (supporting), investigation (supporting), writing – review and editing (supporting). **Arunima Sen:** conceptualization (lead), funding acquisition (lead), methodology (lead), project administration (lead), supervision (lead), writing – review and editing (supporting).

## Funding

The research leading to these results received funding from the Norwegian Financial Mechanism 2014–2021 under the project “Arctic SGD (Arctic Submarine Groundwater Discharge)”, project no. 2019/34/H/ST10/00645. W.H. also acknowledges the support from the Swedish Research Council (VR) under the project “Cryosphere‐driven submarine groundwater in the Arctic” (Project No: 2021‐04962) and STINT through the project Cryosphere‐driven submarine groundwater discharge across the Scandinavian peninsula since the Last Glacial Maximum (IB2022‐9230).

## Conflicts of Interest

The authors declare no conflicts of interest.

## Data Availability

The data used in this study are available at: https://doi.org/10.15468/rkc9pk.
